# Racial and Ethnic Differences in Labor Duration and Cesarean Indications Among Low-Risk Nulliparous Term Singleton Vertex Births: A Retrospective Analysis

**DOI:** 10.3390/jcm15062418

**Published:** 2026-03-21

**Authors:** Elizabeth Mollard, Huijun Xiao, James Bena, Constance Cottrell, Maeve Hopkins

**Affiliations:** 1 College of Nursing, University of Nebraska Medical Center, 985330 Nebraska Medical Center, Omaha, NE 68198-5330, USA; 2 Department of Quantitative Health Sciences, Cleveland Clinic, 9500 Euclid Avenue, Cleveland, OH 44195, USA; 3 Office of Nursing Research and Innovation, Cleveland Clinic, 9500 Euclid Avenue, Cleveland, OH 44195, USA; 4 Division of Maternal–Fetal Medicine, Cleveland Clinic Lerner College of Medicine, Cleveland Clinic, 9500 Euclid Avenue, Cleveland, OH 44195, USA

**Keywords:** primary cesarean, labor progression, labor induction, racial disparities, nulliparous birth

## Abstract

**Background/Objectives:** Racial and ethnic disparities in cesarean birth and labor management persist in the United States, including among individuals considered low risk. Understanding variation in labor progression and cesarean indications within low-risk nulliparous, term, singleton, vertex (NTSV) births may help clarify potential contributors to inequities. This study examined differences in cesarean rates, cesarean indications, and labor duration by race and ethnicity in a low-risk NTSV cohort. **Methods:** We conducted a retrospective secondary analysis of electronic medical record data from 13,231 low-risk NTSV births within a Midwestern academic health system. Multivariable logistic regression models were used to evaluate the likelihood of cesarean birth and cesarean indications by race and ethnicity, adjusting for maternal age, gestational age, body mass index, insurance type, and labor onset. Linear regression models examined differences in first-stage, second-stage, and total labor duration. Interaction terms assessed whether associations varied by labor onset. **Results:** The overall cesarean rate was 29%. Absolute cesarean rates were higher among non-Hispanic Black and Hispanic individuals compared with non-Hispanic White individuals; however, these differences were not statistically significant after adjustment. Labor duration differed significantly by race and ethnicity. Non-Hispanic Black and Hispanic individuals experienced longer median first-stage and total labor durations compared with non-Hispanic White individuals; however, second-stage duration was markedly shorter among non-Hispanic Black individuals. Among induced labors resulting in cesarean birth, non-Hispanic Black and Hispanic individuals had increased odds of cesarean for early arrest of dilation, although these findings should be interpreted as hypothesis-generating, given data limitations in labor onset documentation. Body mass index was positively associated with likelihood of cesarean. **Conclusions:** In this low-risk NTSV cohort, adjusted cesarean rates did not differ significantly by race or ethnicity; however, differences in labor duration and cesarean indication were observed. These findings underscore the importance of continued investigation into labor management practices and structural contributors to obstetric inequities.

## 1. Introduction

The cesarean birth rate in the United States (US) stands at 32%, significantly surpassing the 10–15% rate recommended by the World Health Organization [1,2]. Cesarean rates beyond 10% have not been shown to decrease maternal or neonatal mortality and are associated with heightened maternal risks, including hemorrhage, infection, and complications in subsequent pregnancies [3,4]. Specifically, cesarean birth is associated with 2.7 times the risk of severe maternal morbidity compared to vaginal birth and is associated with approximately 37% of severe maternal morbidity cases [5].

Nulliparous individuals account for one third of births in the US each year and are the major driver of the overall cesarean rate [2]. Around 26% of nulliparous individuals will have a primary cesarean, and nearly 87% of those primary cesareans are followed by a subsequent repeat cesarean in future pregnancies [2]. Safely reducing primary cesarean is an important strategy to reduce the overall cesarean rate and associated maternal morbidity and mortality risks [6]. In this context, efforts to reduce cesarean births focus on avoiding procedures that are not medically indicated while preserving timely use of cesarean as a safe and essential intervention when clinically needed. As such, the American College of Obstetricians and Gynecologists (ACOG) and Society for Maternal and Fetal Medicine (SMFM) created guidelines in 2014, reaffirmed in 2023, titled “Safe Prevention of the Primary Cesarean Delivery” to promote a reduction in non-medically indicated primary cesarean [6].

Racial and ethnic disparities play a significant role in cesarean birth. Maternal health disparities disproportionately affect racial and ethnic minoritized groups, with non-Hispanic Black individuals facing the highest risk, regardless of other health or socioeconomic factors [7,8,9,10,11]. Black, Hispanic, and other individuals from racial and ethnic minoritized groups consistently undergo cesarean at higher rates than White individuals [12,13]. Among low-risk NTSV births, the cesarean rate for Black individuals is 29.5%, compared to 24.6% for White individuals [13]. Furthermore, Black individuals who have a cesarean appear to have an increased risk of related complications compared to White individuals [14]. In a study focusing on low-risk NTSV birth, Black and Hispanic individuals were 21% and 26% more likely to have a cesarean than White individuals [15]. Similarly, a secondary analysis of the nuMoM2b dataset demonstrated persistent racial and ethnic disparities in unplanned cesarean birth among healthy nulliparous individuals [16].

Race is a social construct rather than a biological determinant of health; therefore, observed differences in cesarean outcomes likely reflect social, structural, and healthcare system factors rather than inherent physiological variation [17]. A case in point would be that of individuals diagnosed with arrest disorders (arrest of labor or arrest of descent). In a US sample of individuals diagnosed with an arrest disorder, Black individuals were less likely than White individuals to be given the opportunity to reach complete (10 cm) dilation, reflecting possible differences in intrapartum management and decision-making [12]. In contrast, a prospective cohort study in Nigeria and Uganda documented longer time allowances during spontaneous labor, underscoring that labor management norms vary by practice context and clinical setting [18]. These findings raise questions about whether labor progression and cesarean indications may differ across racial and ethnic groups within comparable clinical populations.

Cesarean data have been further complicated by the rapid increase in elective induction of labor since the publication of the “A Randomized Trial of Induction Versus Expectant Management” (ARRIVE) trial in August 2018 [19]. This large, multicenter, randomized controlled clinical trial, which found that elective induction of labor in the 39th week of pregnancy reduced cesarean and did not negatively impact maternal or fetal outcomes, has led to widespread change in clinical practice where induced labor is significantly more likely compared to before publication of this study [19,20]. While limited data exists on racial disparities related to labor induction, some data suggest that Black individuals who undergo labor induction have increased odds of cesarean compared to White individuals [21].

While prior studies have documented racial and ethnic disparities in cesarean birth among low-risk NTSV populations, less attention has been given to how labor onset and intrapartum labor progression interact with race and ethnicity to shape cesarean indications within low-risk populations, particularly in the context of increasing induction of labor following the ARRIVE trial [12,15,16]. Understanding whether disparities emerge through differences in labor progression, thresholds for intervention, or patterns of cesarean indication may provide insight into mechanisms through which inequities arise even when overall cesarean rates appear similar after adjustment.

The purpose of this study was therefore to examine racial and ethnic differences in cesarean birth, labor progression, and cesarean indications among low-risk NTSV births within a large academic health system. We additionally evaluated whether these associations varied by labor onset (spontaneous versus induced), with particular attention to differences in cesarean indications associated with induced labor.

## 2. Materials and Methods

### 2.1. Study Design

This was a retrospective, descriptive, secondary data analysis of electronic medical records.

### 2.2. Setting

Data were collected from four labor and delivery units within a large academic health system, all located in Ohio and serving a mix of urban, suburban, and rural communities, including the city of Cleveland. The medical center uses an EMR system (Epic Systems Corporation, Verona, WI, USA), and all facilities use a common patient identifier, allowing for integrated data, including result reporting, regardless of which hospital or clinic the individual seeks care.

#### Participants

After obtaining Institutional Review Board approval, the sample was collected from the medical records of individuals who had an NTSV birth on or after 1 September 2018 (following the publication of the ARRIVE trial) and up to 1 July 2022. The sample for this study included individuals with a low-risk NTSV birth at one of the four labor and delivery units within this health system. Specific inclusion criteria were: nulliparous individuals (Parity = 0); term gestation (≥37 weeks gestation); vertex presentation; singleton pregnancy; no prior uterine scar; afebrile on admission; COVID-19 negative. The exclusion criteria for this study were: multiparous individuals (Parity ≥ 1); preterm gestation (<37 weeks); multiple gestation; non-vertex presentation; uterine scar; medical risk factor (diabetes (pre-existing and gestational); hypertension (pre-existing and pregnancy-related); preeclampsia (including HELLP and eclampsia); thrombo/embolic disease (PE, DVT); rheumatic disease; any malignancy, including leukemia and lymphoma; sickle cell disease; heart failure; kidney disease; AIDS/HIV; fetal anomaly); premature rupture of membranes (PROM); febrile on admission; COVID-19 positive. Individuals with missing race or ethnicity were excluded from the analytic sample. Analyses were conducted using complete case data; no imputation was performed. Other variables with missingness were handled through grouping or exclusion as appropriate for each analysis.

### 2.3. Variables

Labor progression. First-stage labor progression was calculated based on the sum of first-stage labor hours and first-stage labor minutes. A similar calculation was applied to second-stage labor progression. Total labor duration was calculated as the sum of the first-stage and second-stage labor progression. All three labor duration variables are in units of hours. Individuals with first-stage labor progression above the 97.5th percentile were evaluated as potential outliers. Duration of labor at the time of cesarean was calculated as the first stage labor duration in hours for those with arrest of labor or the first and second stage for those with arrest of descent in cesareans.

Labor onset. Labor onset variables were incompletely documented across sites; approximately 7750 of 13,231 patients (58.6%) lacked labor onset data in the EMR. Analyses involving labor onset were restricted to records with non-missing labor onset information (complete-case for those variables) (*n* = 5481). Complete-case analysis was applied rather than multiple imputation, given the extent of missingness; while no universal threshold exists, multiple imputation becomes increasingly unreliable as the proportion of missing data grows, and at over 50% missing, the assumptions required for valid imputation are difficult to satisfy [22]. Individuals were classified into three labor onset categories: spontaneous onset (labor type = “spontaneous onset of labor”), induced onset (labor type = “induced onset of labor” or induction = “yes”), and elective induction (induction = “yes” and elective onset = “yes”, when documented). Because elective versus medically indicated induction was incompletely documented across sites, primary regression analyses evaluated labor onset using a two-level variable (induced versus spontaneous). Elective induction was retained for descriptive reporting where available. Labor onset documentation was also disproportionately missing among cesarean births; therefore, onset-stratified cesarean rates were interpreted cautiously.

Insurance. Individuals with “Medicare” or “Medicaid” contained in the benefit plan name were identified as individuals with “public health insurance”. Individuals with other benefit plan names were identified as individuals with “private insurance”, including those marked as self-pay.

Birth Weight. Birth weight was classified into three groups: low birth weight (LBW) (birth weight < 2500 g), normal birth weight (NBW) (birth weight > 2500 and birth weight < 4000), and high birth weight (HBW) (birth weight > 4000) [23].

Gestational age. Individuals with gestational age = 37 or 38 were identified as “early term”, individuals with gestational age = 39 or 40 were identified as “full term”, and individuals with gestational age = 41 were identified as “late term” [23]. Gestational age at delivery was included as a covariate in multivariable regression models.

Race and Ethnicity. Race and ethnicity were recorded in the electronic medical record based on patient self-identification at the time of admission. Race and ethnicity were treated as two distinct variables. Several race and ethnicity categories included small cell counts that would not support stable multivariable modeling. To preserve model convergence and interpretability, less frequent responses were combined into an ‘Other’ category. Because small cell sizes can produce unstable or nonconvergent logistic regression models, this approach reflects data limitations rather than conceptual assumptions about racial, ethnic, or intersecting identity [24].

For analyses involving race alone, individuals were categorized as Black, White, or Other (the latter including all remaining race categories). For analyses involving ethnicity, mutually exclusive race–ethnicity groups were constructed following reporting conventions used by the Centers for Disease Control and Prevention’s National Center for Health Statistics. Individuals were classified as Non-Hispanic Black (Race = Black and Ethnicity not Hispanic), Non-Hispanic White (Race = White and Ethnicity not Hispanic), Hispanic (Ethnicity = Hispanic), or Other (all remaining combinations). These groupings align with national perinatal surveillance methods and allow for consistent interpretation across models while acknowledging the constraints of the available EMR data [25,26].

### 2.4. Statistical Methods

Demographic categorical and continuous variables were summarized as frequencies and proportions (%) or medians and interquartile ranges (IQR), respectively. Continuous variables were assessed for normality using the Shapiro–Wilk test, histogram plots, and QQ plots, as appropriate. Normally distributed continuous variables were compared using two-sample *t*-tests, and non-normally distributed variables were analyzed using the Wilcoxon rank sum test. Categorical variables were compared using Pearson’s chi-squared test or Fisher’s exact test, as appropriate. The Benjamini and Hochberg method was applied to adjust for multiple comparisons where appropriate.

To evaluate differences between racial groups in the likelihood of cesarean birth by labor onset and labor progression, multivariable logistic regression models were constructed. Models included interaction terms between race and labor onset and between race and labor progression, respectively. Covariates determined a priori by investigators, including maternal age, gestational age at delivery, body mass index (BMI), and insurance type, were included in all models. Analyses were conducted using complete case data; no imputation was performed.

Multiple linear regression models were constructed to examine racial differences in first-stage labor duration (time from 0 to 10 cm), second-stage duration (time from 10 cm to birth), and total labor duration. These models included the interaction between race and labor onset to assess whether differences in labor duration varied by labor onset and were adjusted for the same covariates listed above. Multivariable linear regression models were used to assess whether racial and ethnic differences in labor duration persisted after adjustment for covariates. Unadjusted medians and interquartile ranges are reported descriptively in Section 3.4 to characterize the distribution of labor duration across groups.

To examine racial differences in indication for cesarean, multinomial logistic regression models were used. A second multinomial logistic regression model included the interaction between labor induction and race to determine whether induction status modified the association between race and cesarean indication. Results are presented as odds ratios with 95% confidence intervals. Adjustment for multiple testing was performed using the Benjamini and Hochberg procedure. The same analytic approach, including model structure, covariates, interaction terms, and correction procedures, was applied to ethnicity-based analyses.

Model diagnostics were evaluated using residual and influence plots to assess model fit and assumptions. Statistical significance was defined as a two-sided α < 0.05. Data were managed and analyzed using R, version 4.2 (RStudio, Boston, MA, USA).

## 3. Results

Our study included 13,231 low-risk NTSV births. Demographic and individual characteristics between race (Black, White, Other) and ethnicity (Non-Hispanic Black, Non-Hispanic White, Hispanic) groups are summarized in Table 1 and Table 2. The presence of significant differences between these groups in specific characteristics is noted using *p*-values.

### 3.1. Likelihood of Cesarean Birth

The overall cesarean rate was 29%. For every unit increase in Body Mass Index (BMI), there was a 5% increase in the odds of having a cesarean (*p* < 0.001). Those with public health insurance were 0.75 times less likely to have a cesarean compared to those privately insured (*p* = 0.028). Individuals with induced labor had lower odds of cesarean compared to those with spontaneous labor (OR 0.67; 95% CI 0.50–0.91; *p* = 0.028).

When looking at race, rates of cesarean were similar between races but slightly higher among Black (32%) and Others races (31%) when compared to White race (28%). When looking at race and ethnicity, both non-Hispanic Black (31%) and Hispanic (34%) individuals experienced higher rates of cesarean when compared to non-Hispanic White individuals (28%). These differences were not statistically significant after adjustment for covariates.

### 3.2. Indication for Cesarean Birth

The most common indication for cesarean in the overall sample was fetal distress (48%), followed by arrest of descent (23%). Significant variations were noted across racial groups in cesarean indications (*p* < 0.001). Non-Hispanic Black individuals had lower odds of cesarean for arrest of descent compared to Non-Hispanic White individuals (*p* < 0.001). Non-Hispanic Black individuals also had lower odds of cesarean for early dilation arrest (OR 0.05; 95% CI: 0.04–0.08; *p* < 0.001). However, among individuals undergoing induction, Non-Hispanic Black individuals had higher odds of early dilation arrest compared to Non-Hispanic White individuals (OR 21.3; 95% CI: 14.2–31.9; *p* < 0.001). Hispanic individuals undergoing induction had higher odds of early dilation arrest compared to Non-Hispanic White individuals (OR 2.54; 95% CI: 1.25–5.18; *p* = 0.010). Notably, zero cases of arrest before 6 cm were documented among births with documented spontaneous labor onset.

Because complete separation in the spontaneous group precluded direct comparison, a supplementary analysis was conducted to corroborate the disparities within induced labor, comparing odds of cesarean for arrest <6 cm among induced births only across the full cohort without restriction to births with documented labor onset. Non-Hispanic Black individuals undergoing induction had significantly higher odds of arrest <6 cm compared to Non-Hispanic White individuals (OR 2.04; 95% CI: 1.07–3.89; *p* = 0.04), and Hispanic individuals showed a directionally consistent but non-significant elevation (OR 2.38; 95% CI: 0.99–5.73; *p* = 0.06), with the latter likely reflecting limited power among the smaller Hispanic induced subcohort. To contextualize these findings, Table 3 presents the distribution of births by labor onset and race/ethnicity alongside cohort-level cesarean rates and the frequency of cesareans attributed to arrest of dilation <6 cm within induced labor.

### 3.3. Likelihood of Cesarean Birth by Labor Onset

Labor-onset analyses were conducted in the subset with documented labor onset. Because elective versus medically indicated induction status was incompletely documented across sites, regression analyses compared induced labor (inclusive of elective and medically indicated inductions) with spontaneous labor. A reduced likelihood of cesarean was noted in individuals with induced onset compared to spontaneous onset (*p* = 0.04), with no interaction effects between race and labor onset (*p* = 0.17). There were no differences in cesarean by race or ethnicity, regardless of the type of labor onset (*p* = 0.83 (racial analysis), *p* = 0.77 (ethnicity analysis)).

### 3.4. Labor Progress by Race and Ethnicity

The following results describe unadjusted median labor durations by race and ethnicity; multivariable linear regression confirmed that these differences remained statistically significant after adjustment for maternal age, gestational age, BMI, insurance type, and labor onset, and adjusted estimates are reported alongside medians where available.

Black and Other races had a larger median length of the first stages of labor duration compared to White individuals (<0.001). When examined by race, the longest median durations were experienced by Black individuals with a median of 11.57 h, compared to Other race (10.41 h) and White race (8.98 h). After adjustment, Black individuals had a significantly longer first-stage duration compared to White individuals (β = +3.5 h; 95% CI: 0.67–6.2; *p* = 0.033). When examined by ethnicity, non-Hispanic Black individuals had the longest median first-stage duration (11.50 h), followed by Hispanic individuals (11.38 h), compared with non-Hispanic White individuals (8.93 h). After adjustment, non-Hispanic Black individuals had a significantly longer first-stage duration compared to non-Hispanic White individuals (β = +3.2 h; 95% CI: 0.37–6.0; *p* = 0.037).

While race was not a significant predictor of cesarean likelihood relative to the duration of the first stage of labor (*p* = 0.45), an increase in the first stage of labor was associated with increased likelihood of cesarean (*p* = 0.002). In the general sample, for every hour increase in the first stage of labor, the likelihood of cesarean increased by 1% (Adjusted OR: 1.01).

There were statistically significant differences in the total duration of labor by racial groups (*p* < 0.001), with White individuals having the shortest total labor length (median = 10.73 h), compared to Other race (12.12 h) and Black individuals (12.68 h). After adjustment, Black individuals had a significantly longer total labor duration compared to White individuals (β = +3.2 h; 95% CI: 0.38–6.0; *p* = 0.037). Regarding ethnicity, non-Hispanic Black (12.63 h) and Hispanic (12.57 h) individuals had longer total labor durations than non-Hispanic White individuals (10.73 h; *p* < 0.001). After adjustment, non-Hispanic Black individuals had a significantly longer total labor duration compared to non-Hispanic White individuals (β = +2.9 h; 95% CI: 0.08–5.8; *p* = 0.037).

Second-stage duration also differed significantly by race and ethnicity (*p* < 0.001). Black individuals had a markedly shorter median second-stage duration (0.60 h) compared to White individuals (1.38 h). After adjustment, non-Hispanic Black individuals had a significantly shorter second-stage duration compared to non-Hispanic White individuals (β = −0.40 h; 95% CI: −0.65 to −0.14; *p* < 0.001).

## 4. Discussion

National guidelines from ACOG emphasize the importance of safely reducing primary cesarean birth by focusing on clinical practices that may lead to non-medically indicated cesarean birth [6]. These recommendations highlight the need to better understand factors that contribute to variation in cesarean use within low-risk populations. This study explored racial and ethnic disparities in cesarean rates, cesarean indication, and labor progress among 13,231 low-risk NTSV births.

In this cohort, all racial and ethnic groups had higher absolute cesarean rates than non-Hispanic White individuals, although these differences were not statistically significant once we adjusted for confounding variables. These findings should be interpreted with caution due to the retrospective study design and reliance on EMR data.

This analysis was restricted to low-risk births and did not include individuals with medical comorbidities or obstetric complications, which contribute to population-level disparities [7]. By focusing exclusively on low-risk births, the analysis excluded individuals with elevated obstetric risk related to underlying medical conditions compounded by systemic racism and social inequities, which may have increased their likelihood of cesarean birth. Additionally, the overall NTSV cesarean rate within this sample exceeded the national average, which may have masked group-level differences and reduced our ability to detect disparities. Importantly, these findings do not contradict well-documented population-level disparities in maternal morbidity and mortality, which extend beyond mode of delivery and were not directly assessed in this analysis. Rather, the pattern of longer labor durations and differences in cesarean indications within a low-risk cohort suggests that inequities may be expressed through intrapartum processes even when overall adjusted cesarean rates do not differ.

However, even in this limited-risk cohort, significant differences in labor progression were observed. Labor durations were significantly longer for all racial and ethnic groups compared to non-Hispanic White individuals, with non-Hispanic Black individuals experiencing the longest median duration. An additional finding that warrants consideration is the substantially shorter second-stage duration observed among non-Hispanic Black individuals compared with non-Hispanic White individuals (median 0.60 h vs. 1.38 h). At first glance, this appears counterintuitive given the longer first-stage durations observed in the same group. However, non-Hispanic Black individuals in this cohort also had lower odds of cesarean birth for arrest of descent, suggesting that once complete dilation was achieved, progression to vaginal birth was generally successful. This pattern is not unique to the present cohort and has been interpreted in prior research as reflecting variation in clinical management rather than intrinsic physiologic differences [27,28]. Taken together, these findings suggest that the decision points contributing to cesarean birth for non-Hispanic Black women may occur earlier in labor progression, particularly during the first stage and in the context of induction. Non-Hispanic Black individuals who reach complete dilation appeared to have high success rates of achieving vaginal birth in our study, underscoring the importance of allowing adequate time for labor progression before diagnosing arrest disorders in this population.

Race and ethnicity are social constructs that reflect lived experiences within social and healthcare systems rather than biological determinants. Observed differences in labor progression should therefore be interpreted within broader structural and clinical contexts rather than as evidence of inherent physiological variation [12,18]. The physiologic impact of stress, anxiety, and exposure to racism, both within healthcare settings and society at large, has been shown to affect labor physiology and may contribute to longer labor durations [29,30,31]. In one study, fear of childbirth was the strongest predictor of prolonged labor, a factor that may be intensified among individuals from racially and ethnically minoritized individuals who must navigate historical inequities along with ongoing and worsening disparities, as reflected in current maternal mortality and morbidity data [30,31,32].

Our findings on differences in labor progress by race and ethnicity, combined with previous research, raise questions about how labor norms are defined and applied in clinical practice. Variation in intervention thresholds and clinical management practices may contribute to differences in cesarean indication across populations [33]. Additionally, clinical practice is often guided by published ‘labor curves’ that estimate dilation from 6 to 7 cm will take approximately 2.2 h at the 95th percentile, yet one study of Black birthing individuals found a median of 4.9 h for the same progression [6,18,34]. Interpreted within a framework that understands race as a social rather than biological construct, these findings suggest that more individuals of all racial and ethnic backgrounds may be able to safely complete labor when provided adequate time and support. These findings underscore the importance of ongoing evaluation of labor management standards across diverse populations.

Beyond labor duration, racial and ethnic variations in indications for cesarean were observed. Comparable variation in primary cesarean indications by race and ethnicity has been reported within a national birth center registry [35]. The ARRIVE trial has led to a surge in elective inductions. Our findings align directionally with ARRIVE in that induction was not associated with increased cesarean likelihood in this cohort; however, interpretation differs because our comparison group was spontaneous labor rather than expectant management. There were no statistically significant differences in the overall cesarean rate by race or ethnicity when labor was induced. However, when labor was induced and a cesarean was performed, non-Hispanic Black and Hispanic individuals had a markedly increased risk of cesarean for early arrest of dilation (<6 cm). These findings suggest that labor onset and management practices may interact with race and ethnicity in complex ways and warrant further investigation [21].

Consistent with prior literature, higher BMI was associated with increased odds of cesarean delivery; each one-unit increase in BMI was associated with a 5% increase in odds [36]. Obesity is shaped by broader social conditions, including food access, environmental stress, and the cumulative effects of racism, and these factors may be relevant when interpreting cesarean risk in racially and ethnically diverse samples [36]. Future research should continue to explore how BMI interacts with race and ethnicity in relation to cesarean birth [36].

The association between public insurance and lower cesarean odds was an unexpected finding that warrants cautious interpretation. Hospital unit was not included as a covariate in the primary models, and the four delivery units within our health system differ in payer mix and clinical practice patterns, so site-level confounding cannot be excluded. It is also possible that this finding reflects differences in care processes associated with Medicaid coverage, including differences in prenatal care utilization, provider continuity, or the clinical thresholds applied during labor management for publicly versus privately insured patients. Prior work has documented that privately insured patients may face higher rates of non-medically indicated cesarean, driven in part by patient preference, scheduled delivery, and provider incentive structures, which could contribute to an apparent protective effect of public insurance in a model that does not account for indication type [37,38,39]. The relationship between insurance type, institutional practice patterns, and cesarean decision-making is complex and warrants dedicated investigation.

Future research should incorporate prospective designs and multi-level analyses to better understand how patient characteristics, provider decision-making, and institutional practices contribute to observed differences [40,41]. Application of the Intrapartum Cesarean Delivery Classification System (ICDCS) in future work would allow more granular characterization of the specific pathways through which labor arrest leads to cesarean birth across racial and ethnic groups [42].

### Limitations

This retrospective secondary analysis of EMR data limits causal inference and the ability to control for unmeasured confounding. Generalizability is also limited because the sample was drawn from a single academic health system, and regional differences in patient populations, institutional practices, and health policy may affect applicability to other settings.

Race was self-reported, and ethnicity data were inconsistently captured in the EMR, which limited precision in some categories [43]. Due to data constraints, race and ethnicity categories were aggregated, which may have obscured more granular or intersectional identities. Several variables, including insurance, were broadly grouped and may have masked within-category variation.

The low-risk inclusion and exclusion criteria further limit generalizability to the broader obstetric population. Labor onset was incompletely documented in the EMR for approximately 59% of the cohort; complete-case analysis was therefore applied to models incorporating labor onset, which may limit generalizability if missingness was systematically related to labor characteristics or outcome. We were unable to reliably distinguish elective from medically indicated induction or characterize the indication for induction (e.g., post-dates), which may have introduced residual confounding in analyses involving labor onset. The OR of 21.3 for arrest of dilation before 6 cm among induced Non-Hispanic Black individuals should be interpreted as hypothesis-generating, given the complete separation in the logistic model arising from zero spontaneous labor cases with this indication.

In addition, provider role during intrapartum management was not reliably documented, and key labor management variables (e.g., oxytocin dosing patterns, adequacy of uterine activity/MVUs, timing and frequency of cervical exams, and standardized criteria for arrest diagnoses) were not consistently extractable across sites. Although multiple-testing corrections were applied, the number of comparisons increases the possibility of Type I error. Finally, EMR-based quantitative data cannot capture patient perceptions of care or experiences of mistreatment. These limitations should be considered when interpreting the findings.

## 5. Conclusions

This retrospective analysis of 13,231 low-risk NTSV births identified significant differences in labor duration and cesarean indications across racial and ethnic groups within a single academic health system. Although adjusted overall cesarean rates did not differ significantly by race or ethnicity, longer labor durations and variation in cesarean indications suggest that inequities may manifest through intrapartum processes even within clinically low-risk populations. Cesarean likelihood was also associated with BMI, insurance status, and labor onset. These findings should be interpreted in the context of EMR-based measurement constraints, incomplete documentation of induction indication, and limited availability of granular labor management variables. Prospective, multisite studies incorporating detailed intrapartum management data and standardized diagnostic criteria are needed to clarify mechanisms underlying variation in labor progression and cesarean indications and to inform equity-centered practice improvement.

## Figures and Tables

**Table 1:** Demographic and individual characteristics summary comparing between Black, White, and Others racial groups.

Characteristic	Overall, *n* = 13,231 ^1^	Black, *n* = 1993 ^1^	Others, *n* = 1382 ^1^	White, *n* = 9856 ^1^	*p*-Value ^2^
Age	31.37 (27.10, 34.65)	26.44 (23.30, 30.49) ^ab^	30.95 (25.62, 34.72) ^ac^	32.04 (28.56, 35.12) ^bc^	<0.001
BMI	27.98 (24.04, 33.30)	30.12 (25.49, 36.02) ^ab^	26.95 (23.56, 31.64) ^ac^	27.77 (23.92, 32.92) ^bc^	<0.001
Use of vacuum	1086 (11%)	142 (10%)	132 (13%)	812 (11%)	0.044
Reason for Cesarean		^ab^	^a^	^b^	<0.001
Fetal Distress	696 (48%)	142 (61%)	88 (50%)	466 (45%)	
Arrest < 6 cm	199 (14%)	41 (18%)	20 (11%)	138 (13%)	
Arrest > 6 cm	222 (15%)	36 (15%)	29 (17%)	157 (15%)	
Arrest of Descent	327 (23%)	15 (6.4%)	38 (22%)	274 (26%)	
Mode of birth		^ab^	^ac^	^bc^	<0.001
Operative Vaginal Delivery	1311 (10.0%)	129 (6.5%)	155 (11%)	1027 (10%)	
Spontaneous Vaginal Delivery	8032 (61%)	1223 (62%)	793 (58%)	6016 (61%)	
Cesarean	3823 (29%)	626 (32%)	428 (31%)	2769 (28%)	
Length of 1st stage of labor (h)	9.47 (4.83, 16.25)	11.57 (5.70, 20.55) ^ab^	10.41 (5.49, 18.90) ^ac^	8.98 (4.63, 15.17) ^bc^	<0.001
Length of 2nd stage of labor (h)	1.25 (0.62, 2.33)	0.60 (0.32, 1.18) ^ab^	1.29 (0.63, 2.54) ^ac^	1.38 (0.72, 2.47) ^bc^	<0.001
Total duration of labor (h)	11.14 (6.38, 17.98)	12.68 (6.65, 21.55) ^ab^	12.12 (7.13, 20.65) ^ac^	10.77 (6.28, 17.07) ^bc^	<0.001
Labor onset		^b^		^b^	0.020
Spontaneous Onset	802 (15%)	145 (18%)	87 (16%)	570 (14%)	
Induced Onset	3736 (68%)	560 (68%)	387 (69%)	2789 (68%)	
Elective Induced	943 (17%)	122 (15%)	87 (16%)	734 (18%)	
Insurance status		^ab^	^ac^	^bc^	<0.001
Private Insured	10,040 (76%)	810 (41%)	904 (65%)	8326 (84%)	
Public Health Insurance	3191 (24%)	1183 (59%)	478 (35%)	1530 (16%)	
Neonatal birth weight		^b^	^c^	^bc^	<0.001
LBW	429 (3.4%)	105 (5.6%)	62 (4.6%)	262 (2.7%)	
NBW	11,487 (90%)	1701 (90%)	1208 (90%)	8578 (90%)	
HBW	841 (6.6%)	75 (4.0%)	68 (5.1%)	698 (7.3%)	
Gestational age		^b^		^b^	<0.001
early term	3381 (26%)	598 (30%)	369 (27%)	2414 (24%)	
full term	8374 (63%)	1223 (61%)	871 (63%)	6280 (64%)	
late term	1476 (11%)	172 (8.6%)	142 (10%)	1162 (12%)	
Duration of labor at time of Cesarean (h)	11.93 (6.92, 19.77)	16.75 (8.20, 29.25) ^b^	15.93 (8.65, 23.45) ^c^	11.58 (6.07, 17.97) ^bc^	0.002

^1^ Median (IQR); *n* (%). ^2^ Kruskal–Wallis rank sum test; Pearson’s Chi-squared test; Fisher’s exact test. Benjamini & Hochberg method was applied for multiple pairwise comparison adjustment. a: Significant difference between Black and Others groups; b: Significant difference between Black and White groups; c: Significant difference between Other and White groups.

**Table 2:** Demographic and individual characteristics summary comparing between Hispanic, Non-Hispanic Black, Non-Hispanic White, and Others racial groups.

Characteristic	Overall, *n* = 13,231 ^1^	Hispanic, *n* = 686 ^1^	Others, *n* = 990 ^1^	Non-Hispanic Black, *n* = 1965 ^1^	Non-Hispanic White, *n* = 9590 ^1^	*p*-Value ^2^
Age	31.37 (27.10, 34.65)	26.90 (23.60, 32.02) ^abc^	32.24 (28.02, 35.16) ^ce^	26.44 (23.33, 30.49) ^ade^	32.10 (28.68, 35.17) ^bd^	<0.001
BMI	27.98 (24.04, 33.30)	29.34 (25.05, 35.88) ^bc^	26.34 (23.04, 29.86) ^cef^	30.10 (25.44, 36.01) ^de^	27.74 (23.91, 32.80) ^bdf^	<0.001
Use of vacuum	1086 (11%)	35 (7.5%) ^bc^	117 (17%) ^cef^	139 (10%) ^e^	795 (11%) ^bf^	<0.001
Reason for Cesarean		^bc^	^ce^	^de^	^bd^	<0.001
Fetal Distress	696 (48%)	57 (61%)	58 (47%)	138 (61%)	443 (44%)	
Arrest < 6 cm	199 (14%)	11 (12%)	12 (9.8%)	40 (18%)	136 (14%)	
Arrest > 6 cm	222 (15%)	17 (18%)	19 (15%)	35 (15%)	151 (15%)	
Arrest of Descent	327 (23%)	8 (8.6%)	34 (28%)	15 (6.6%)	270 (27%)	
Mode of birth		^bc^	^cef^	^de^	^bdf^	<0.001
Operative Vaginal Delivery	1311 (10.0%)	42 (6.1%)	141 (14%)	125 (6.4%)	1003 (11%)	
Spontaneous Vaginal Delivery	8032 (61%)	413 (60%)	541 (55%)	1211 (62%)	5867 (61%)	
Cesarean	3823 (29%)	230 (34%)	302 (31%)	614 (31%)	2677 (28%)	
Length of 1st stage of labor (h)	9.47 (4.83, 16.25)	11.38 (5.81, 19.06) ^b^	10.32 (5.47, 18.46) ^f^	11.50 (5.66, 20.49) ^d^	8.93 (4.62, 15.10) ^bdf^	<0.001
Length of 2nd stage of labor (h)	1.25 (0.62, 2.33)	0.98 (0.48, 1.90) ^abc^	1.48 (0.70, 2.80) ^ce^	0.60 (0.32, 1.18) ^ade^	1.38 (0.73, 2.48) ^bd^	<0.001
Total duration of labor (h)	11.14 (6.38, 17.98)	12.57 (6.88, 20.62) ^b^	12.15 (7.29, 20.50) ^f^	12.63 (6.61, 21.47) ^d^	10.73 (6.27, 17.01) ^bdf^	<0.001
Labor onset		^bc^	^c^	^d^	^bd^	<0.001
Spontaneous Onset	802 (15%)	56 (21%)	50 (13%)	142 (17%)	554 (14%)	
Induced Onset	3736 (68%)	183 (67%)	276 (70%)	558 (68%)	2719 (68%)	
Elective Induced	943 (17%)	33 (12%)	68 (17%)	119 (15%)	723 (18%)	
Insurance status		^abc^	^cef^	^ade^	^bdf^	<0.001
Private Insured	10,040 (76%)	362 (53%)	731 (74%)	801 (41%)	8146 (85%)	
Public Health Insurance	3191 (24%)	324 (47%)	259 (26%)	1164 (59%)	1444 (15%)	
Neonatal birth weight		^ab^	^f^	^ad^	^bdf^	<0.001
LBW	429 (3.4%)	32 (4.8%)	51 (5.4%)	102 (5.5%)	244 (2.6%)	
NBW	11,487 (90%)	590 (88%)	858 (90%)	1683 (91%)	8356 (90%)	
HBW	841 (6.6%)	47 (7.0%)	44 (4.6%)	69 (3.7%)	681 (7.3%)	
Gestational age		^a^		^ad^	^d^	<0.001
early term	3381 (26%)	175 (26%)	268 (27%)	592 (30%)	2346 (24%)	
full term	8374 (63%)	424 (62%)	627 (63%)	1204 (61%)	6119 (64%)	
late term	1476 (11%)	87 (13%)	95 (9.6%)	169 (8.6%)	1125 (12%)	
Duration of labor at time of Cesarean (h)	11.93 (6.92, 19.77)	19.05 (7.26, 23.48)	15.90 (9.58, 23.73)	16.75 (8.20, 27.56)	11.58 (6.07, 17.78)	0.003

^1^ Median (IQR); *n* (%). ^2^ Kruskal–Wallis rank sum test; Pearson’s Chi-squared test. Benjamini & Hochberg method was applied for multiple pairwise comparison adjustment. a: Significant difference between Hispanic and non-Hispanic Black groups; b: Significant difference between Hispanic and non-Hispanic White groups; c: Significant difference between Hispanic and Others groups; d: Significant difference between non-Hispanic Black and non-Hispanic White groups; e: Significant difference between non-Hispanic Black and Others groups; f: Significant difference between non-Hispanic White and Others groups.

**Table 3:** Classification of Births and Cesarean Delivery by Labor Onset Group and Race/Ethnicity, with Arrest of Dilation Indication Among NTSV Births (*N* = 13,231).

Racial/Ethnic Group	Group 1 (Spontaneous Onset) *N* Births	Group 2a (Induced Onset) *N* Births	Total Cohort *N* Births	Total CS *N* (%)	Arrest < 6 cm Group 2a Only ^†^ *N* (% of Coded CS)
Non-Hispanic White	554	3442	9590	2677 (27.9%)	136 (14%)
Non-Hispanic Black	142	677	1965	614 (31.2%)	40 (18%)
Hispanic	56	216	686	230 (33.5%)	11 (12%)
Other	50	344	990	302 (30.5%)	12 (9.8%)
Total	802	4679	13,231	3823 (28.9%)	199 (14%)

Group 1: spontaneous onset of labor. Group 2a: induced onset of labor, inclusive of both medically indicated induction (*n* = 3736) and elective induction (*n* = 943). N births for Groups 1 and 2a reflect the subset with documented labor onset (*n* = 5481; 41% of cohort); labor onset documentation was incomplete and non-randomly missing across sites. ^†^ This column is to provide descriptive context for the distribution of early dilation arrest across labor onset groups and racial/ethnic categories. Arrest of dilation <6 cm (early dilation arrest) as a cesarean indication is reported exclusively within Group 2a because zero cases were documented among births with spontaneous onset of labor, resulting in complete separation in models restricted to births with documented labor onset. Accordingly, all such cesarean indications are therefore attributable to the induced onset group, and resulting estimates are inflated. Supplementary analyses using the full induced cohort demonstrated more moderate but persistent elevations in odds among Non-Hispanic Black and Hispanic individuals. Percentage denominator is cesarean births with a coded indication documented in the electronic medical record. Total *N*, total cesarean *N*, and overall cesarean rate reflect the full analytic cohort (*N* = 13,231) and are consistent with values reported in manuscript Table 1 and Table 2. Race/ethnicity categories follow CDC/NCHS reporting conventions. NTSV = nulliparous, term, singleton, vertex.

## Data Availability

Datasets used in this study are not available for use due to protected health information.

## Abbreviations

The following abbreviations are used in this manuscript:

NTSV	Nulliparous Term Singleton Vertex
